# Supplementary material for: Transcriptomic Insights into Mechanisms of Early Seed Maturation in the Garden Pea (Pisum sativum L.)
Source: Cells. 2020 Mar 23;9(3):779. doi: 10.3390/cells9030779 (PMC7140803; doi:10.3390/cells9030779)

Sprint−2,10(3)

Global Summary

%DE = 0.29

genes with fdr < 0.2 = 184 (94 + / 90 −)

genes with fdr < 0.1 = 162 (83 + / 79 −)

genes with fdr < 0.05 = 162 (83 + / 79 −)

genes with fdr < 0.01 = 137 (69 + / 68 −)

genes in genesets = 0

<FC> = 0

<t−score> = 0

<p−value> = 0

<fdr> = 0.71

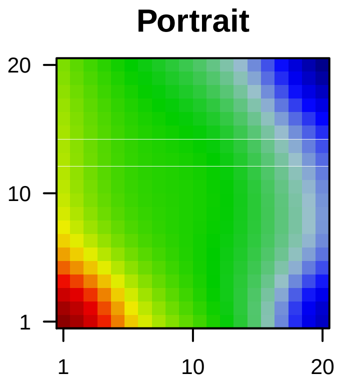

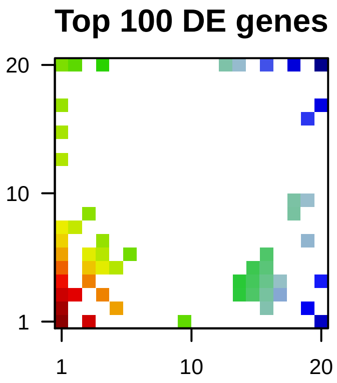

Global Genelist

Rank	ID	log(FC)	p−value	fdr	Metagene	Description
1	TRINITY_DN1	0.67	2e−16	1e−15	1 x 2	
2	TRINITY_DN3	0.24	2e−16	1e−15	3 x 5	
3	TRINITY_DN1	−13.29	2e−16	1e−15	20 x 1	
4	TRINITY_DN1	−8.1	2e−16	1e−15	20 x 20	
5	TRINITY_DN5	−0.23	2e−16	1e−15	2 x 20	
6	TRINITY_DN3	0.2	2e−16	1e−15	1 x 15	
7	TRINITY_DN3	0.19	2e−16	1e−15	1 x 17	
8	TRINITY_DN2	−3.15	2e−16	1e−15	20 x 1	
9	TRINITY_DN2	−1.59	2e−16	1e−15	20 x 1	
10	TRINITY_DN3	1.05	2e−16	1e−15	1 x 1	
11	TRINITY_DN3	−0.74	2e−16	1e−15	20 x 17	
12	TRINITY_DN3	−0.25	2e−16	1e−15	19 x 10	
13	TRINITY_DN5	0.69	2e−16	1e−15	1 x 1	
14	TRINITY_DN4	4.64	2e−16	1e−15	1 x 1	
15	TRINITY_DN4	−0.46	2e−16	1e−15	18 x 20	
16	TRINITY_DN1	0.63	2e−16	1e−15	1 x 1	
17	TRINITY_DN3	−1.32	2e−16	1e−15	20 x 1	
18	TRINITY_DN2	0.56	2e−16	1e−15	1 x 2	
19	TRINITY_DN2	−0.94	2e−16	1e−15	20 x 20	
20	TRINITY_DN2	0.72	2e−16	1e−15	1 x 1	

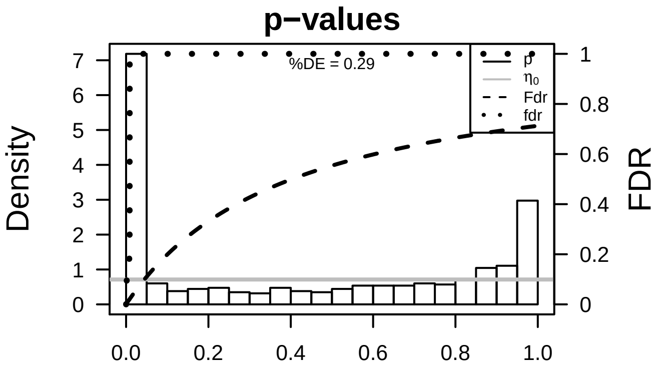

Supplement: Supplementary file 1 [file cells-09-00779-s001.zip › Supplementary materials/FileS3.zip!/FileS2/Hormones - Results/Summary Sheets - Samples/Sprint.2.10.3..pdf]
